# Spatial Scale Gap Filling Using an Unmanned Aerial System: A Statistical Downscaling Method for Applications in Precision Agriculture

**DOI:** 10.3390/s17092106

**Published:** 2017-09-14

**Authors:** Leila Hassan-Esfahani, Ardeshir M. Ebtehaj, Alfonso Torres-Rua, Mac McKee

**Affiliations:** 1 Civil & Environmental Engineering, Utah State University, Logan, UT 84322, USA; mac.mckee@usu.edu; 2 College of Science & Engineering, University of Minnesota, Minneapolis, MN 55455, USA; ebtehaj@umn.edu; 3 Utah Water Research Laboratory, Utah State University, Logan, UT 84322, USA; a.torres@aggiemail.usu.edu

**Keywords:** Landsat, UAV, downscaling, NDVI, soil moisture, precision agriculture

## Abstract

Applications of satellite-borne observations in precision agriculture (PA) are often limited due to the coarse spatial resolution of satellite imagery. This paper uses high-resolution airborne observations to increase the spatial resolution of satellite data for related applications in PA. A new variational downscaling scheme is presented that uses coincident aerial imagery products from “AggieAir”, an unmanned aerial system, to increase the spatial resolution of Landsat satellite data. This approach is primarily tested for downscaling individual band Landsat images that can be used to derive normalized difference vegetation index (NDVI) and surface soil moisture (SSM). Quantitative and qualitative results demonstrate promising capabilities of the downscaling approach enabling effective increase of the spatial resolution of Landsat imageries by orders of 2 to 4. Specifically, the downscaling scheme retrieved the missing high-resolution feature of the imageries and reduced the root mean squared error by 15, 11, and 10 percent in visual, near infrared, and thermal infrared bands, respectively. This metric is reduced by 9% in the derived NDVI and remains negligibly for the soil moisture products.

## 1. Introduction

Precision agriculture (PA) is the art and science of using advanced computational and remote sensing technologies to produce overall gains in profitability and environmental stewardship. PA relies on airborne and satellite remote sensing to support adaptive management practices for different areas within agricultural fields based on varying soil types, landscape, water demand, and crop stresses [1,2]. Passive airborne remote sensing observations have been used to estimate several agricultural variables, such as soil moisture [3,4], chlorophyll [5], crop types [6], disease types [7], yield [8], and irrigation water demand [9]. In this study, we focus on downscaling the visible to near-infrared satellite imageries and the derived normalized difference vegetation index (NDVI), and surface soil moisture (SSM) estimates as agricultural variables of interest.

NDVI has been used extensively in several agricultural studies, and high-resolution soil moisture data are essential for understanding the dynamics and productivity of hydrologic [10,11] and agricultural [12] systems. In the past few decades, space-borne remote sensing of soil moisture has progressed significantly through the launch of several satellites, including the Soil Moisture and Ocean Salinity (SMOS) satellite by the European Space Agency (ESA), and the Soil Moisture Active Passive (SMAP) satellite by NASA. A major advantage of remote sensing in microwave bands is that soil moisture can be measured regardless of time of day or atmospheric conditions. However, while these soil moisture data provide unique opportunities to better understand mesoscale land atmosphere interactions, they are incompatible to provide information at scaled resolve scales that are useful in the context of agricultural management or ecological modeling [13]. As a result, research devoted to increasing the spatial resolution of satellite soil moisture data [3,14,15] has led to the developments of a variety of spatial downscaling methods.

A range of techniques of varying complexities has been developed for downscaling. Merlin et al. [16] developed an empirical radiative transfer model to downscale the near-surface soil moisture data by a factor of 8 and reported a 3% reduction in the mean squared error. In a more recent study, [10] they improved the spatial resolution of a passive microwave-derived product from a resolution of approximately 40 km to approximately 4 km using a disaggregation method. This product used MODIS 1 km resolution data to provide more accurate soil moisture data for a range of agricultural and hydrologic applications. Similar research by Choi and Hur [17] improved the spatial representation of soil moisture products using disaggregation approaches and MODIS data. Chauhan et al. [18] developed an approach to evaluate soil moisture at a resolution of about one kilometer by applying optical/infrared data to passive microwave derived soil moisture data at a resolution of about 25 km. In that study, NDVI was used to improve soil moisture downscaling by applying the “Universal Triangle” approach.

Most of these studies have focused on developing microwave soil moisture downscaling methods that provide soil moisture products at a resolution of a few kilometers. However, this resolution is still too coarse for agricultural applications. Here, we propose a downscaling method that aims to increase the resolution of Landsat imagery by a factor of 2 to 4 to produce improved derivative products, such as soil moisture or NDVI.

The proposed downscaling model relies on super-resolution techniques that use sparse approximation methods, which have shown advantages in enhancing the resolution of precipitation fields [19]. Succinctly stated, sparse approximation methods allow us to better recover high-frequency features that have been lost as a result of the smoothing effects of a low-resolution sensor. The super resolution method needs a joint library of low- and high-resolution imagery that can be used to train the downscaling scheme. To this end, we use coincident observations of the low-resolution Landsat imageries (RGB, NIR, Thermal Infrared) and the derived agricultural (NDVI and SM) products at resolution 30 m and spectral data products derived from high-resolution airborne imageries at 0.15-m resolution by the AggieAir platform (AggieAir, 2016) to populate the low- and high-resolution libraries.

The paper is organized as follows. Section 2 introduces the study area and the remote sensing platforms used. The proposed downscaling scheme and calibration of the raw data for calculation of the derivative products are also explained in this section. Section 3 presents the results of the downscaling scheme for individual spectral bands, NDVI and SM products. Section 4 discusses the concluding remarks and points out to potential future directions.

## 2. Materials and Methods

### 2.1. Study Area

**Figure 1:**
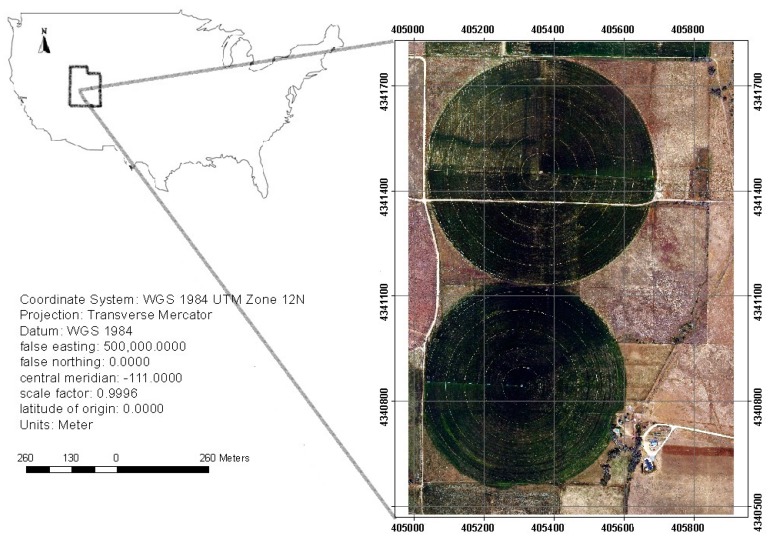
The location of the study area in Utah, United States.

The study area includes two agricultural fields located in Scipio, Utah, USA, centered at 39°14′ N 112°6′ W with the area of 1.35 km^2^ (Figure 1). Oats and alfalfa are the main crops, grown from May to September, which are irrigated by modern center pivot sprinkler systems. This study was based on data collected on 1 June, 9 June, and 17 June 2013, a time frame that covers a growing cycle for alfalfa. Figure 1 shows the natural color images of the field on 1 June.

### 2.2. AggieAir and Landsat

AggieAir is a collection of remote sensing platforms and payloads developed at the Utah Water Research Laboratory at Utah State University [20]. These completely autonomous unmanned aerial vehicles (UAVs) were programmed to navigate over the study area and capture images in the blue, green, red, and near infrared (NIR) spectra at 0.15 m resolution and in the thermal band at about 0.6 m resolution. The wavelength range peaks at around 420, 500, 600, and 800 nm, respectively, for the blue, green, red, and NIR bands. The thermal images were down-processed to 0.15 m resolution to achieve resolutions consistent with other AggieAir products [21]. In order to study and compare AggieAir and Landsat data, flight experiments were conducted to coincide with Landsat overpasses (on the same dates and at about 18:04 scene center time). In addition, the experiments were designed to cover the maximum variation of land cover as a result of crop growth stage. Therefore, the first growing cycle of alfalfa, from emergence to harvest, was selected for the experiments. This growing cycle was covered by four Landsat overpasses; however, one overpass was removed from the experimentation plan due to cloud cover (24 May). The UAV flew over the study area on 1 June, 9 June, and 17 June 2013.

### 2.3. Landsat and AggieAir Reflectance Homogenization

**Figure 2:**
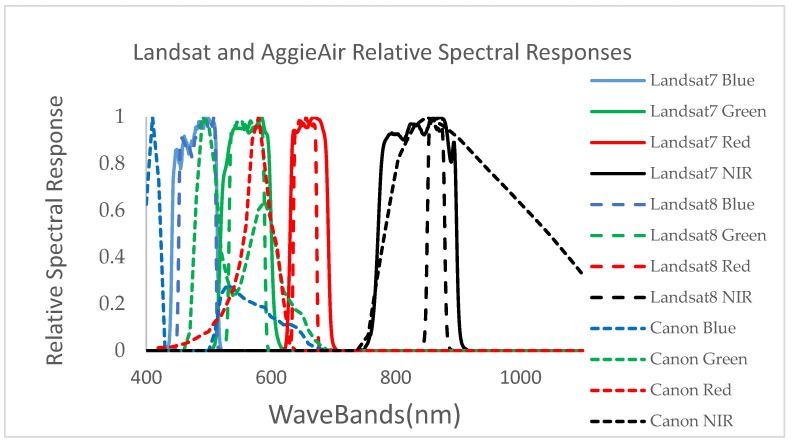
The spectral responses of AggieAir Canon cameras versus Landsat sensors (the spectral response is scaled for all individual bands from 0 to 1).

The visible camera used in the AggieAir flights is a Canon S-95 (with a 10-megapixel charge-coupled device (CCD) sensor with an International Standards Organization (ISO) range of 80–3200 [5]. NIR imagery was captured with the same camera modified to replace the manufacturer’s optical filter with a Wratten 87 NIR filter that allows NIR wavelengths of 750 nm and larger. The relative spectral responses of the VIS–NIR cameras were obtained using the algorithm provided by Jiang et al. [22]. AggieAir also carries a small, low-power, microbolometer thermal camera from Infrared Cameras Inc. (ICI) [23] (Infrared Cameras Incorporated, 2012). The AggieAir and Landsat VIR spectral responses are shown in Figure 2.

Figure 2 shows that the spectral responses of the AggieAir cameras are by no means identical to those of the Landsat sensors. Moreover, the significance of atmospheric contamination in the observations of the two sensors is drastically different. These differences often lead to some systematic differences between the observations by the two sensors. For potential biases, a de-biasing (bias fixing) step was conducted prior to downscaling. To that end, we assumed that the Landsat data represent the baseline product for bias correction and applied the bias correction to the high-resolution imagery acquired from the UAV platform.

**Figure 3:**
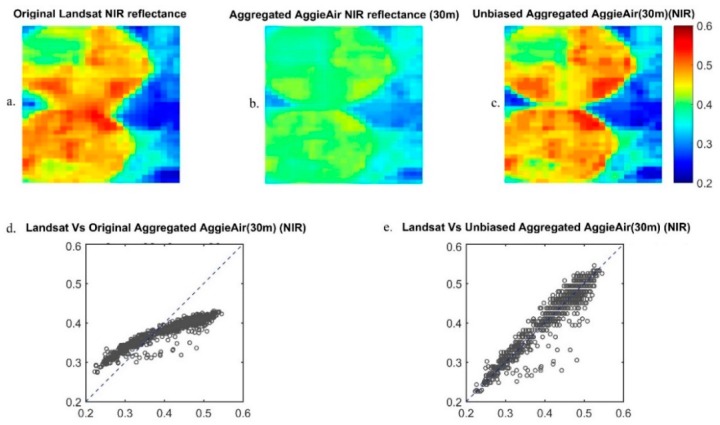
(**a**) Original Landsat NIR reflectance image; (**b**) aggregated AggieAir NIR image (resolution: 30 m); (**c**) Unbiased aggregated AggieAir NIR image (resolution: 30 m); (**d**) Scatterplot of Landsat versus the original aggregated AggieAir NIR image; (**e**) Scatter lot of Landsat versus the unbiased aggregated AggieAir NIR image.

Given the spectral characteristics of the Cannon cameras (Figure 2) used in the study (Figure 1), it is evident that 30-m aggregated AggieAir imagery was not congruent at the pixel level with the Landsat reflectance standard product (from Landsat 7 ETM+ and Landsat 8 OLI). Using USGS-developed Landsat Point Spread Function information for each of the Landsat 7 and 8 bands (USGS, 2015 personal communication), AggieAir reflectance bands were aggregated up to become compatible with the 30-m resolution Landsat imagery over the study area. We used histogram matching techniques for bias correction [24] on AggieAir imagery (Figure 3). This technique uses a probability matching function that adjusts the histogram of the deviated image from the AggieAir platform to match the histogram of the reference Landsat imageries.

Figure 3a shows the original Landsat NIR band surface reflectance for 1 June 2013 over the study area, and Figure 3b shows the aggregated NIR band reflectance image from AggieAir. Figure 3c shows the unbiased AggieAir NIR reflectance image at 30-m resolution with histogram matching applied. Figure 3d,e demonstrate the effectiveness of this approach with pixel-level scatter plots of the original and unbiased aggregated AggieAir NIR band versus the corresponding Landsat NIR band at a resolution of 30 m.

### 2.4. Downscaling Individual Spectral Bands

To downscale the Landsat data using coincident high-resolution AggieAir observations, we conducted downscaling experiments on all Landsat red/green/blue and infrared (VIR) bands with scaling factors of 2 and 4. This corresponds to the 15 and 7.5 m spatial resolution grids, respectively. 

As previously noted, several downscaling methods have been used for agricultural applications. Here, we focus on a super-resolution approach that has been successfully implemented for downscaling remote sensing data [19]. The method relies on a sparse approximation technique that allows us to recover high-resolution imagery from low-resolution counterparts by solving an inverse problem. In lieu of solving a large-scale inverse problem for the entire field of observation, the method focuses on small-scale constitutive elements of the observation field that consist of a few nearby pixels, often called a patch. In other words, the entire low-resolution observation field is broken down into patches, and then the method seeks to obtain their high-resolution counterparts through solving small-scale inverse problems that are computationally tractable. This method relies on a set of training examples that implicitly encodes the correspondence between the high- and low-resolution patches. These training examples are collected in two matrices called low- and high-resolution dictionaries. The method attempts to reconstruct the high-resolution patches with a few patches in the low-resolution dictionary by using a sparse approximation technique. It then assumes that the low- and high-resolution fields are geometrically similar and, thus, uses the same coefficients to combine the training patches in the high-resolution dictionary.

Specifically, let us assume that an *m*-by-*n* low-resolution (LR) field of the Landsat imagery is denoted by $X_{l}\in R^{m\times n}$ (e.g., 30-m grid), while its high-resolution (HR) counterpart from the AggieAir imagery is $X_{h}\in R^{sm\times sn}$ (i.e., 15 m or 7.5 m with downscaling factors, respectively, *s* = 2 and 4). Let us assume that the low-resolution images can be upscaled to the same size as the high-resolution images using an interpolation operator, denoted by $I_{s}\left(X_{l}\right)\in R^{sm\times sn}$. Consequently, we can obtain a residual field as $X_{r}=X_{h}-I_{s}\left(X_{l}\right)$, which contains the lost information between the high- and low-resolution fields that we wish to recover through the downscaling approach using a limited number of coincident low- and high-resolution images from Landsat and AggieAir. The optimal estimation of the residual field was carried out on a patch-wise basis, as previously described, to avoid a cumbersome optimization problem. The downscaling uses a large number of training examples of LR and HR patches to reconstruct the residual field. Let us assume that those patches are vectorized and stored in column space of the low- $D_{l}=\left[x_{l1},x_{l2},\;\ldots .\;,x_{lM}\right]\in R^{\;q\;\times \;M}$ and high-resolution $D_{h}=\left[x_{h1},x_{h2},\ldots .\;,x_{hM}\right]\in R^{\;s^{2}q\;\times \;M}$ dictionaries. For a given low-resolution image, the method breaks it down into N vectorized low-resolution patches $Y_{l}={\left\{y_{li}\in R^{q\times l}\right\}}_{i=1}^{N}$ . Each patch is estimated with a linear combination of a few patches in the LR dictionary (i.e., column vectors of $D_{l}$). In this study, a Greedy method called orthogonal matching pursuit (OMP) by Mallat and Zhang [25] has been used. Initializing the estimation residual by the observed signal, at each iteration, first the support of the representation coefficients is updated by selecting an atom which has the maximum inner product with the estimation residual and then, given the support set, the values of the representation coefficients are being updated through an ordinary least squares. The iterations continue until a certain number of atoms is selected or the magnitude of the estimation residual falls below a certain threshold [19].

### 2.5. Developing Agricultural Variables

To demonstrate application of the proposed downscaling scheme for use in precision agriculture, NDVI and soil moisture (SM) products are derived from the downscaled original VIR imagery at resolutions of 15 and 7.5 m.

For SM estimations, original Landsat spectral imagery and the downscaled counterparts were fed into a previously developed soil moisture retrieval model [26]. The adapted method relies on Bayesian artificial neural network (Bay-ANN) algorithms that learn the soil moisture variability using a set of training examples of inputs (either the normal 30 m resolution Landsat imagery or downscaled imagery) and outputs (soil moisture). To provide the outputs, an intensive ground sampling program was conducted over the study area during the Landsat overpasses. The data collection procedure was designed to cover the maximum spatial distribution of soil moisture with respect to the irrigation system function, crop type, and soil texture characteristics [26,27].

## 3. Results and Discussion

### 3.1. Downscaled Spectral Bands

Imagery over the study area in the red, green, blue, NIR, and thermal spectral bands was used to study the quantitative performance of the proposed downscaling scheme. The scheme requires the development of a dictionary training set, i.e., a representative set of coincidental pairs of low- and high-resolution patches of images. The model restored the optimal high-resolution estimate using supervised learning from 8766 training patches obtained from three pairs of high- and low-resolution images from Landsat low- and the AggieAir high-resolution observations on 1 June, 9 June, and 17 June 2013. Hereafter, two different scenarios are defined; in the first scenario, all high and low resolution counterparts from three flight dates were used to populate the dictionary and, in the second scenario, one pair at a time is left out for validation purposes.

In the first scenario, all three pairs of high- and low-resolution images (all 8766 patches) were used to train the downscaling model. The downscaled products were compared with bias-corrected high-resolution AggieAir products as our benchmark. In the second scenario, the model was trained with two pairs (out of the three available high- and low-resolution pairs), and one pair was retained for testing.

**Figure 4:**
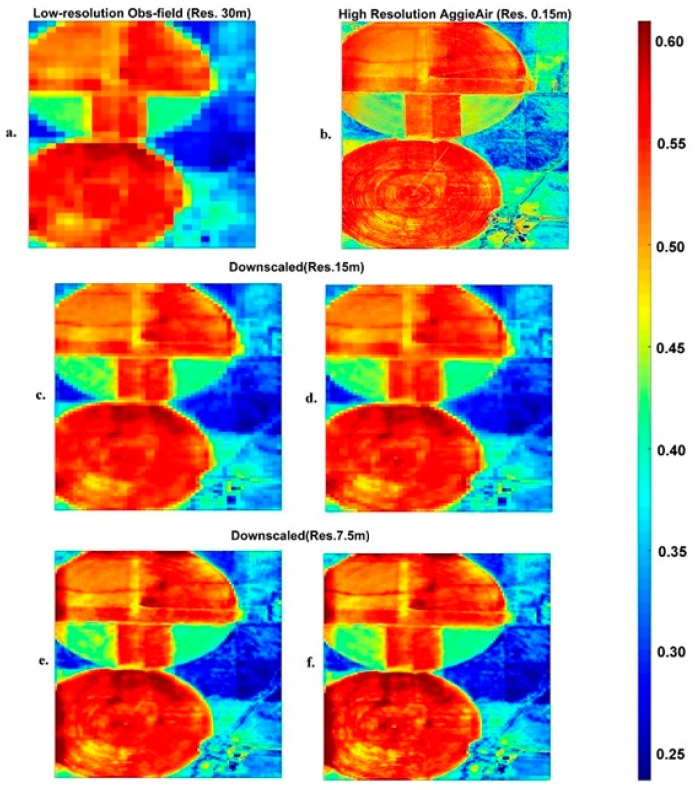
(**a**) Low-resolution observation (Landsat NIR); (**b**) high-resolution observation (AggieAir NIR); (**c**,**e**) downscaled NIR at 15 and 7.5 m resolution, respectively (first scenario); (**d**,**f**) downscaled NIR 15 and 7.5 m resolution, respectively (second scenario).

To illustrate the effectiveness of the downscaling model in the first scenario, the 30 × 30 m low-resolution Landsat images (individual bands of red, green, blue, NIR, and thermal infrared) of the study area were downscaled to 15 and 7.5 m grid sizes. Figure 4 depicts the low-resolution NIR band imagery from Landsat (Figure 4a) acquired on 1 June 2013 at about 18:04 center time (UTC time), and its high-resolution counterpart from the AggieAir platform (Figure 4b). Figure 4c shows the NIR image downscaled from 30 m to the finer resolution of 15 m using the downscaling scheme. The results demonstrate how well the learning procedure performed and how some high frequency features can be recovered. The same procedure was repeated for a scale factor of 4, as shown in Figure 4e. The results show that suitable information in the training set leads to an improved algorithm success. As long as the model can find very close or identical pairs of patches in the dictionary, it can successfully generate downscaled products. Figure 4d,f show the results from the same downscaling scheme for scale factors of 2 and 4 where the high resolution patches of interest were not part of the training dataset (second scenario).

**Figure 5:**
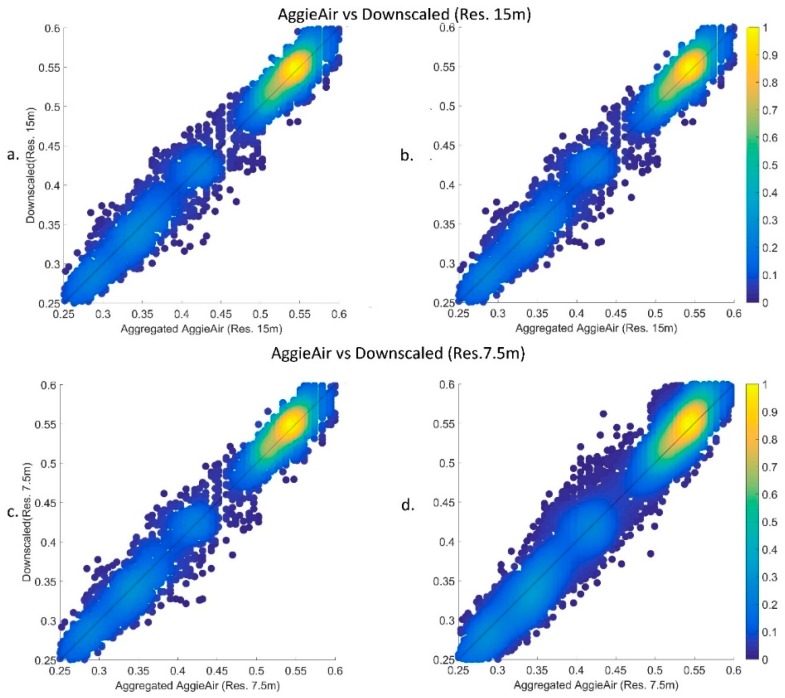
Pixel-wise comparison of AggieAir imagery versus downscaled NIR images as at resolution 15 and 7.5 m for the first (**a**,**c**) and second (**b**,**d**) scenarios, respectively.

The differences between the downscaling results from the above two scenarios are illustrated in Figure 5 on a pixel-wise basis, comparing reflectance values of aggregated imagery from AggieAir with downscaled imagery resulting from our model. AggieAir images are aggregated to reach the same resolution as the downscaling results. This step has been carried out to achieve spatial consistency and make it feasible to perform a pixel-wise comparison. Figure 5 shows the one-by-one scatter plots of the downscaled fields from Landsat vs the upscaled field from AggieAir. Figure 5a,b show the scatter plots of the downscaled Landsat NIR field at scale factor of 2 (commensurate with resolutions of 15 m), versus the upscaled AggieAir image at the same resolution. The same plots are shown in Figure 5c,d for a scale factor of 4. The image of interest was included in the training dataset (first scenario) for the results shown in Figure 5a,c, while the information of the image of interest was not included in training examples (second scenario) for the results shown in Figure 5b,d. The downscaling quality metrics are reported in Table 1. These matrices are presented as percentage values that show the relative reduction in root mean square error as a quality metric. Note that the comparisons are performed after the biased correction approach was applied. The metrics show that the differences between the downscaled and benchmark images have decreased significantly in most of the cases.

As is evident from model statistics and a visual inspection of Figure 4d,f and Figure 5b,d, the effectiveness of the model slightly degrades when the information of the downscaled image is not among the training examples. However, the decrease in accuracy is not significant, which confirms the generalization capability of the proposed method. This claim can be quantitatively supported by the information reported in Table 1, as the improvement of root mean squared values are mostly remained in the range of 10 ± 5 percent. Clearly, the degradation in downscaling quality increases as the downscaling level increases and the recovered high-resolution image becomes more blurred.

Figure 4 and Figure 5 present the imagery of one representative date (1 June 2013) out of the three available dates, and Table 1 shows the quality matrices for all three dates in the experiment. Although the goodness-of-fit statistics do not noticeably depend on the scaling factor, the blurriness of the images in Figure 4e,f indicate degradation in the results for larger scaling factors. Table 1 shows that the downscaling models function very similarly for both scenarios in terms of qualitative and quantitative performance and the RMSE values show that the overall quality of the downscaling model has not significantly deteriorated for larger scaling factors.

Since the model learns from both scale levels, the resulting map conveys the information from both ends of Landsat and AggieAir. In Figure 4, the field exterior area is shown by lower values of NIR (less reflective) and more dark-reddish color (more reflective) on the upper side of the southern field that has come from the Landsat product. At lower scales (finer resolution), the influences of the AggieAir products become more visible. Visual inspections show that the color gradient of the downscaled fields tends to be closer to the AggieAir product, which demonstrates that the model is able to find and properly use relevant matches of high- and low-resolution patches in the dictionaries.

### 3.2. Remotely-Sensed Agricultural Variables Derived from Downscaled Bands

In order to test the downscaling scheme presented here for agricultural applications, the downscaled observations in VIR bands were used to infer high-resolution NDVI and soil moisture fields at resolutions of 15 and 7.5 m. NDVI was considered as a direct derivative of the spectral bands, having a diverse application in agricultural studies, which can be derived through a simple relationship between the red and NIR bands. Surface soil moisture (SSM) was considered as the indirect derivative of individual downscaled bands. As previously noted, here we use the model by Hassan-Esfahani et al. [27] to drive high resolution (0.15 m resolution) soil moisture products for the fields. NDVI and SSM were computed at high resolution (0.15 m) from AggieAir products, low resolution (30 m) from Landsat products, and downscaled levels (15 and 7.5 m resolution), and the resulting maps were compared versus aggregated AggieAir products as the baseline. Table 2 provides the goodness-of-fit statistics for these direct and indirect agricultural products versus aggregated AggieAir products. It is important to note that for the soil moisture product a model error is involved, which adds to the downscaling error. In other words, the low level of improvements in downscaled soil moisture products might be due to the insensitivity of soil moisture model to small subgrid variability of the downscaled spectral fields.

Table 2 shows that the downscaling model does not significantly degrade in derivation of high-resolution NDVI products compared to its performance for individual spectral bands with respect to the used quality measures. The goodness-of-fit matrices did not significantly degrade as the scaling level changed, nor when the image of interest was excluded from the training set. Here again the improvement in root mean squared values stayed in the range of 10 ± 5 percent which is absolutely comparable with the information in Table 1. The highest improvement is associated with the overpass in 9 June 2013. This improvement reflects higher association between Landsat 8 spectral response compatibility with AggieAir technology products. This compatibility drags less systematic error from data collection procedure and data homogenization step to further analysis.

Table 2 shows the degradation of results when the modeling is performed for indirect products (SSM). As presented here, the percent of improvement ratio noticeably decreases for almost one decimal point while comparing NDVI versus SSM models. One reason for this condition could be the fact that the downscaling error propagated and increased as a result of the nonlinear soil moisture Bayesian model. In addition, for direct NDVI products, we have compared observational data with same nature but from different sources while, for indirect products (SSM), we have compared modeling outputs, which may contain significant additive model errors.

The results suggest that the degree of improvements is dependent on the observed fields and perhaps it physical conditions such as vegetation coverage and soil moisture variability. The results from the second flight show the highest quality among the three sets of data. This holds true for the individual spectral bands and the direct and indirect products. For this flight date, there were fewer visible heterogeneities and the coarse-scale images from Landsat 8 did not require use of interpolation methods, as did those of Landsat 7, to fill missing data. Further development of the methodology and further availability of the AggieAir data provides the opportunity to extend the method for downscaling of the satellite data. However, the downscaling model performance is highly tied to numbers and the diversity of sample patches that are available in the dictionaries. In other words, more high-resolution counterparts that are being provided in the dictionaries create a higher quality trained reference dataset that can be applied for improved downscaling.

## 4. Conclusions

This study presents a statistical approach to compare remotely sensed agricultural variables at different spatial scales in qualitative and quantitative terms. The comparison is based on a new downscaling scheme that can add detailed spatial information to a low-resolution fields. The downscaling scheme was tested for individual spectral bands acquired from a variety of different sensors (Landsat 7, 8, and AggieAir). Tests were also conducted for direct and indirect derivatives of the imagery that have information value to agricultural management.

The downscaling algorithm uses a modern statistical learning method and can recover the lost higher frequency details in spectral bands and their direct and indirect derivatives. The method learns from a representative set of training data from low- and high-resolution patches by solving a constrained optimization problem.

The results presented here suggest future studies to explore the generality and performance of the methodology and its practical implications for use in applications such as precision agriculture.

Since the high-resolution imagery and derivative agricultural products are not available in many practical cases, these results are instructive as a quantitative evaluation of downscaling success rate for similar cases.

In future studies, the range of applicability and effectiveness of the downscaling methodology presented here should be precisely determined for larger datasets. The method should also be empirically tested for its performance on different direct and indirect products as well. This flexible methodology could also facilitate studies using Earth observation data from SSM missions, such as AMSR-E, SMOS.

## Figures and Tables

**Table 1:** The root mean squared error (RMSE) quantifying the quality of the downscaling scheme for different scaling factors for all spectral bands.

Spectral Band	Downscaling Level	Including in the Training Set	RMSE		
			1 June 2013	9 June 2013	17 June 2013
			Improvement Ratio	Improvement Ratio	Improvement Ratio
Red	2	YES	14%	16%	15%
		NO	8%	11%	10%
	4	YES	25%	27%	24%
		NO	17%	20%	13%
Green	2	YES	14%	13%	15%
		NO	7%	8%	15%
	4	YES	25%	26%	23%
		NO	13%	15%	10%
Blue	2	YES	13%	15%	12%
		NO	5%	8%	5%
	4	YES	23%	29%	20%
		NO	9%	14%	9%
NIR	2	YES	13%	20%	16%
		NO	5%	10%	6%
	4	YES	14%	23%	5%
		NO	6%	14%	2%
Thermal	2	YES	9%	7%	6%
		NO	1%	1%	2%
	4	YES	0.12%	0.16%	0.16%
		NO	0.07%	0.09%	0.06%

**Table 2:** Root mean squared error (RMSE) for testing the performance of downscaling scheme in derivation of direct (NDVI) and indirect (SSM) agricultural products. High-resolution AggieAir products are considered as the reference.

Agricultural Product	Downscaling Level	Including in the Training Set	RMSE		
			1 June 2013	9 June 2013	17 June 2013
			Improvement Ratio	Improvement Ratio	Improvement Ratio
NDVI	2	YES	10%	15%	12%
		NO	5%	11%	12%
	4	YES	10%	11%	8%
		NO	7%	7%	6%
SSM	2	YES	1.81%	1.67%	1.54%
		NO	1.32%	1.42%	1.43%
	4	YES	1.49%	1.14%	1.78%
		NO	1.23%	1.12%	1.08%
